# Effects of *Salvia miltiorrhiza* active compounds on placenta-mediated pregnancy complications

**DOI:** 10.3389/fcell.2023.1034455

**Published:** 2023-01-13

**Authors:** Jingyin Kong, Songjun Li, Yingting Li, Min Chen

**Affiliations:** ^1^ Department of Prenatal Diagnosis and Fetal Medicine, The Third Affiliated Hospital of Guangzhou Medical University, Guangzhou, China; ^2^ Department of Reproduction Medical Center, The Third Affiliated Hospital of Shenzhen University, Shenzhen, China

**Keywords:** placental pregnancy complications, *Salvia miltiorrhiza*, active compounds, placental development, preeclampsia

## Abstract

Placenta-mediated pregnancy complications (PMPCs), including preeclampsia (PE), fetal growth restriction (FGR), and recurrent spontaneous abortion (RSA), occur in approximately 5% of pregnancies and are caused by abnormal placenta development. The development of effective therapies for PMPCs is still challenging due to the complicated pathogenesis, such as disrupted vascular homeostasis and subsequent abnormal placentation. Synthetic drugs have been recommended for treating PMPCs; however, they tend to cause adverse reactions in the mother and fetus. *Salvia miltiorrhiza* (*S. miltiorrhiza*) has potential effects on PMPCs owing to its advantages in treating cardiovascular disorders. *S. miltiorrhiza* and its active compounds could attenuate the symptoms of PMPCs through anticoagulation, vasodilation, antioxidation, and endothelial protection. Thus, in this review, we summarize the literature and provide comprehensive insights on *S. miltiorrhiza* and its phytochemical constituents, pharmacological activities, and on PMPCs, which would be valuable to explore promising drugs.

## 1 Introduction

The human placenta, a specialized organ that mediates exchanges between the mother and fetus, is essential for a successful pregnancy and fetal health. Its development begins during the implantation of the blastocyst (Hemberger et al., 2020). Chorionic villi, as structural and functional units of the placenta, are consisted of two layers of trophoblasts (James et al., 2022). The inner layer is composed of proliferative villous cytotrophoblasts (vCTBs), which can differentiate into outer layer villous syncytiotrophoblasts that form a physical barrier to pathogens (Turco and Moffett, 2019). Cytotrophoblast cells invade the maternal spiral arteries and replace the maternal endothelium. The remodeling of maternal spiral arteries reduces the resistance of blood flow to meet the nutrition transport for fetus (Turco and Moffett, 2019; James et al., 2022). Defective trophoblast differentiation and function cause incomplete spiral artery remodeling, contributing to PMPCs (Staff et al., 2022). However, the physiopathological mechanism of PMPCs has yet to be elucidated (Freitag et al., 2020; Wu et al., 2020; Staff et al., 2022).

PMPCs result in high maternal and neonatal morbidity rates as aforementioned (Kuwabara et al., 2020). Synthetic drugs have been recommended to treat PMPCs (Chappell et al., 2021). In particular, low-dose aspirin can attenuate the symptoms of PE (Hodgetts Morton and Stock, 2022; Walsh and Strauss, 2022). However, synthetic drugs have adverse reactions in the mother and fetus. In contrast, traditional Chinese medicine (TCM), with a long usage history, has drawn increasing attention in recent years due to its fewer side effects (Yang et al., 2022). Clinicians have started treating PMPCs with TCM compounds, achieving satisfactory therapeutic effects (Zhang et al., 2006).

**FIGURE 1:**
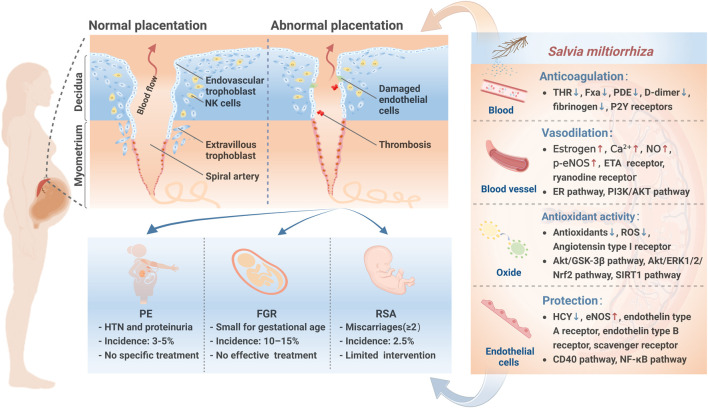
PMPCs pathophysiology and the mechanisms of *S. miltiorrhiza* in PMPCs. During normal placentation, extravillous cytotrophoblasts invade the uterine spiral arteries of the decidua andmyometrium, transforming the spiral arteries into large-caliber vessels. However, during abnormal placentation in PMPCs, failed spiral artery remodeling constrains maternal-fetal interface blood flow, which contributes to the pathogenesis of PMPCs. NK, natural killer; PE, preeclampsia; FGR, fetal growth restriction; RSA, recurrent spontaneous abortion; HTN, hypertension; THR, thrombin; Fxa, factor Xa; NO, nitric oxide; p-eNOS, phosnhorylation of endothelial nitric oxide synthase; ETA, endothelin A; ROS, reactive oxygen species; HCY, homocysteine.

*S. miltiorrhiza*, known as Danshen in Chinese, is a perennial plant of the Lamiaceae family (Zeng et al., 2017). Modern pharmacological studies have found that *S. miltiorrhiza* affects the promotion of blood circulation, modulation of vascular endothelial cells, and reduction of immune interactions in the mother-fetus interface. *S. miltiorrhiza* injection, derived from *S. miltiorrhiza* extract, plays a remarkable role in treating PMPCs (Bai et al., 2019; Chen and Yang, 2019). Herein, we review the active compounds, potential effects, and the pharmacological mechanisms of *S. miltiorrhiza* in PMPCs (Figure 1).

## 2 Active compounds of *S. miltiorrhiza* in PMPCs

*S. miltiorrhiza* shows extensive biological activities, including antioxidant, antibacterial and anti-inflammatory. Thus, it is widely used for the treatment of various diseases, containing hyperlipidemia, stroke, and cardiovascular and cerebrovascular diseases (Chong et al., 2019). *S. miltiorrhiza* is first described in TCM in the *Compendium of Materia Medica *(Bencao Gangmu, Ming dynasty, 1596 AD). The primary bioactive compounds in *S. miltiorrhiza* are devided into two major groups of chemicals (Zhang et al., 2022). One group involves water-soluble phenolics, such as salvianolic acid A (Sal A), salvianolic acid B (Sal B), lithospermic acid and rosmarinic acid (Wang et al., 2019). The other group is consisting of lipophilic compounds, such as tanshinone I, tanshinone IIA (Tan IIA), tanshinone IIB, cryptotanshinone, and dihydrotanshinone I (Wang et al., 2020). Hence, we outline the valuable active compounds of *S. miltiorrhiza* associated with PMPCs, as listed in Table 1.

**TABLE 1:** Active compounds and potential mechanisms of *S. miltiorrhiza* in PMPCs.

Classification	Active compound (Structure)	Potential mechanisms in PMPCs	References
water-soluble phenolics	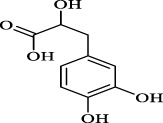 Danshensu	- Inhibit activated platelets	Shen et al. (2011)
		- Scavenge free radical	Zhang et al. (2010); Shen et al. (2011)
		- Protect the endothelial cells	Zhang et al. (2010)
	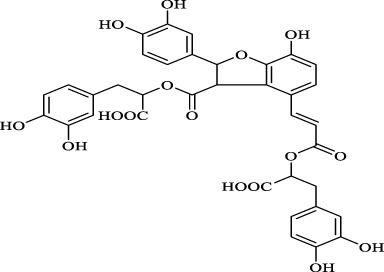 Salvianolic acid B	- Downregulate the expression of PAR-1 and phosphorylation of PKC	Zhang et al. (2021a)
		- Promote the vasodilation through nitric oxide	Shou et al. (2012)
		- Reduce the production of oxygen free radicals	Zhao et al. (2008); Xiao et al. (2020)
		- Regulate the gene expression of antioxidant enzymes	
		- Protect endothelial cells against apoptosis	Liu et al. (2007)
	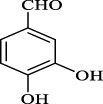 Protocatechualdehyde	- Have anticoagulant activity	Song Wang et al. (2008)
		- Decrease oxidative stress level	Ji et al. (2021)
		- Restore endothelial function	
	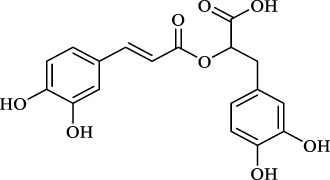 Rosmarinic acid	- Scavenge free radical	Lamaison et al. (1991); Huang and Zheng, (2006)
		- Reduce intracellular ROS	
		- Exhibit anti-angiogenic activity	Huang and Zheng, (2006)
	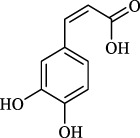 Caffeic acid	- Scavenge superoxide anion radical	Gülçin, (2006)
		- Modulate eNOS expression and phosphorylation	Migliori et al. (2015)
lipophilic tanshinones	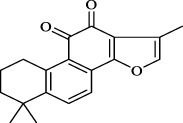 Tanshinone IIA	- Dilate the vessels *via* K^+^ channel	Chan et al. (2011)
		- Inhibit CLIC1 expression level	Zhu et al. (2017)
		- Preserve umbilical vein endothelial cells	Lin et al. (2006)
	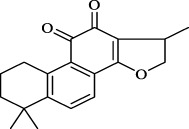 Cryptotanshinone	- Produce vasodilatation *via* decreased Ca^2+^ influx	Lam et al. (2008)
		- Regulate NF-κB and Nrf-2/HO-1 pathways	Wang et al. (2018)
		- Prohibit ET-1 secretion	Zhou et al. (2006)
		- Decrease ET-1 mRNA expression level	

## 3 Effects of *S. miltiorrhiza* in PMPCs

### 3.1 *S. miltiorrhiza* ameliorates PE

PE is a serious condition characterized by hypertension and proteinuria after 20 weeks of pregnancy (Vata et al., 2015), with an incidence rate of 3%–5% and at least 42,000 maternal deaths yearly (Chappell et al., 2021). PE is a severe threat to maternal and fetal health during pregnancy and childbirth and increases the long-term risk of cardiovascular diseases in mothers and their fetuses (Staff, 2019). It is divided into early-onset and late-onset types (Lisonkova and Joseph, 2013). PE presents reduced trophoblast invasion and defective spiral artery remodeling, which triggers a series of pathophysiological processes, such as antiangiogenesis, vascular inflammation and oxidative stress, resulting in systemic endothelial dysfunction and clinical manifestations (Ortega et al., 2022). The blood vessels of a patient with PE narrowed because of impaired trophoblast invasion and incomplete spiral artery remodeling. The placenta is deprived of blood and oxygen, resulting in abnormal placentation (Hong et al., 2021). *S. miltiorrhiza* has been used to treat PE because of its ability to increase blood flow; however, the mechanism is not fully understood (Zhang et al., 2006).

*S. miltiorrhiza* injection upregulates the serum insulin-like growth factor-1 and placental growth factor, which enhances the invasion and migration abilities of placental trophoblastic cells and improves the condition of patients with early-onset PE and the prognosis of the mother and the fetus (Li and Qiu, 2020). After the treatment, the systolic and diastolic blood pressures and 24 h urine protein levels decrease remarkably. *S. miltiorrhiza* also improves vascular endothelial function and ischemia-hypoxia status in patients with PE. And there is, abnormal expression of long-chain non-coding RNA (lncRNA) in the placenta of patients with PE (Liang and Meng, 2021). These lncRNAs may cause changes in the expression of downstream regulatory target genes, thereby reducing the invasion of trophoblasts and leading to uterine spiral artery remodeling disorders (Cheng et al., 2019). *S. miltiorrhiza* injection prevents PE from progressing to severe PE or eclampsia and improves maternal and infant outcomes through downregulating the expression of lncRNAs in the placental tissues (Ma et al., 2021). Furthermore, in PE animal models, *S. miltiorrhiza* injection effectively lower blood pressure and alleviate proteinuria to normal levels by increasing platelet count and reducing thrombomodulin expression in the placenta (Shen et al., 2011; Peng and Huang, 2021). The decrease of endogenous and exogenous coagulation factors, factor Xa (FXa), D-dimer, and fibrinogen indicates that the coagulation in PE rat models improved. And long-term low-dose Sal A administration exerts better efficacy through enhancing anticoagulant activity (Shen et al., 2011). Therefore, *S. miltiorrhiza* injection could effectively ameliorate placenta-related indicators and vascular endothelial function in PE (Jiao et al., 2021; Zhang et al., 2021b).

### 3.2 S. miltiorrhiza mitigates the severity of FGR

FGR is a severe pregnancy-related disease wherein fetuses cannot achieve the expected weight. It is a pivotal cause of stillbirths and an essential factor affecting the long-term health of the fetus. FGR is mainly caused by abnormal placentation due to insufficient placental development and microvascular resistance, which causes blood circulation disorders in 10%–15% of pregnant women (Aplin et al., 2020; Wang et al., 2020; Ortega et al., 2022). It is divided into early-onset and late-onset according to the gestational age at onset. Early-onset FGR occurs before 32 weeks of pregnancy, accounting for 20% of all cases. Late-onset FGR (≥32 weeks) occurs in about 70% of patients and has a weak correlation with hypertension (approximately 10%) (Audette and Kingdom, 2018). FGR can be attributed to maternal (such as malnutrition, hypertension, PE) and fetal factors (such as chromosomal abnormalities and multiple births) and placental dysfunction. However, placental dysfunction is the most frequent underlying cause of FGR (Aplin et al., 2020; Freitag et al., 2020; Melamed et al., 2021). Incomplete invasion of vCTBs results in inhibition of placental growth, impairment of placental function, long-term hypoxia, and fetus malnutrition. The combination of sodium lactate Ringer’s injection and *S. miltiorrhiza* is more effective than sodium lactate Ringer’s injection alone (Wei et al., 2021).

### 3.3 *S. miltiorrhiza* improves the adverse symptoms of RSA

RSA, defined as the failure of two or more clinically recognized pregnancies before 20–24 weeks of gestation, occurs in nearly 2.5% of women trying to conceive. The etiology of RSA is still not fully understood. Trophoblast cells are the most critical cells in placental development, and their proliferation, migration, and invasion are essential for establishing and maintaining a successful pregnancy. Defective trophoblast function impairs uterine spiral artery reconstruction and is implicated in RSA (Wu et al., 2020). Currently, RSA is mainly treated with immunotherapy and anticoagulation, but these therapies have no specificity (Pan et al., 2022).

In RSA mouse models, Tan IIA is ascertained to reduce the rate of embryo loss (Tong et al., 2022). *S. miltiorrhiza* injection has a specific curative effect on patients with RSA through improving the trophoblast cell function and prethrombotic state (Liu et al., 2021). However, relevant studies remain limited and further studies are needed to elucidate the mechanism of *S. miltiorrhiza* in RSA.

## 4 Potential mechanisms of *S. miltiorrhiza* in PMPCs

### 4.1 Anticoagulation

The precise regulation of blood coagulation is critical in maintaining a successful pregnancy (True et al., 2022). The blood coagulation cascade is a complicated process regulated by plasma proteins and cofactors affected by different coagulation factors (Davie, 1995). Human pregnancy involves hemochorial placentation wherein the villous covered by a trophoblast layer is subdivided into functional units bathed by maternal blood, ensuring maternal-fetal exchanges (Kohli et al., 2022). Meanwhile, pregnant women are at high risk of hemorrhage, organ-specific thrombosis, and thromboinflammation (Kohli et al., 2022). Decidual thrombosis and spontaneous intrauterine umbilical artery thrombosis are associated with FGR, placental abruption, PE, and preterm birth (Vedmedovska et al., 2011). Thus, inhibiting coagulation is a promising strategy to treat PMPCs (Boeldt and Bird, 2017). Thrombin (THR), which is closely involved with the occurrence of thrombosis and embolism, and FXa, which is a common mediator of intrinsic and extrinsic coagulation, play crucial roles in the coagulation cascade (Yang et al., 2020). Some components of *S. miltiorrhiza* have been reported in response to these key events. Tan IIA, tanshinone I, dihydrotanshinone I, and cryptotanshinone, act as THR/FXa inhibitors, thereby destroying the coagulation cascade to achieve anticoagulation (Yang et al., 2020). Danshensu, one of the active compounds of *S. miltiorrhiza*, strongly mitigates blood viscosity and increases hematocrit levels due to its antithrombotic and antiplatelet aggregation effects (Yu et al., 2014). Moreover, *S. miltiorrhiza* injection substantially improves coagulation in PE rats (Peng and Huang, 2021). Notably, three major active compounds of *S. miltiorrhiza* (Sal A, B, and C) function by targeting the prothrombotic P2Y1 and P2Y12 receptors (Liu et al., 2018). Sal B inhibits platelet activation by decreasing phosphodiesterase activity and antagonizing the P2Y12 receptors (Liu et al., 2014). Coactivation of both P2Y receptors plays an essential role in ADP-induced platelet aggregation, whereas the inhibition of both receptors has a synergistic effect on antithrombotic therapy (Liu et al., 2018). However, to date, there are currently few commercial drugs targeting the P2Y receptors.

### 4.2 Vasodilation

A successful pregnancy is associated with dramatic changes in the uterine blood flow, facilitating the maternal-fetal exchanges of respiratory gas and meeting the needs of the developing fetus (Bowman et al., 2021). Impaired endothelium-dependent vasodilation has been implicated in the development of PE. Danshensu directly acts on vascular endothelial and smooth muscle cells to promote vascular relaxation (Wang et al., 2013; Lin et al., 2022). It also dilates the vessels and improves blood circulation to increase renal blood flow and improve renal function in PE rats (Peng and Huang, 2021). The mechanisms underlying pregnancy-associated uterine vasodilation are related to increased estrogen receptor (ER) levels, which drive the production of specific ER-dependent vasodilators in the uterine artery (Bai et al., 2020). Tan IIA exerts its vasodilation effect through activating the ER signal pathway and increasing endothelial nitric oxide synthase (eNOS) gene expression level, nitric oxide (NO) production, ERK1/2 phosphorylation, and Ca^2+^ mobilization (Fan et al., 2011). It also promotes vasodilatation by decreasing the expression of the endothelin A receptor, which is a primary receptor in modulating vasoconstriction (Chen et al., 2017). Magnesium acetate, an active compound of *S. miltiorrhiza*, dilates blood vessels through activating the PI3K/AKT pathway and increasing the phosphorylation of eNOS (Liu et al., 2019). Sal B is a potentially effective natural compound to lower blood pressure and alleviate hypertension-associated vascular dysfunctions (Ling et al., 2017). It mediates vasodilation by inhibiting extracellular calcium influx and intracellular calcium release. The calcium release mechanism relies on the ryanodine receptor family, one of the families of calcium release channels (Shou et al., 2012).

### 4.3 Antioxidant

Oxidative stress is widely believed to disrupt the balance between ROS and the antioxidant system (Hussain et al., 2021). During pregnancy, nutritional deficiencies result in adverse offspring outcomes (Sebastiani et al., 2019). Excessive oxidative stress impairs maternal and placental functions by limiting the antioxidant supply and eventually results in the decreased metabolic health of offspring (Schoots et al., 2018; Nadeem et al., 2019; Burton et al., 2021). Cryptotanshinone improves doxorubicin-induced oxidative damage and apoptosis through inhibiting the opening of the mitochondrial permeability transition pore *via* the Akt/GSK-3β pathway (Wang et al., 2021). Danshensu has a protective effect against oxidative stress during ischemia-reperfusion injury through ROS scavenging, and it enhances the activity of endogenous antioxidants, such as superoxide dismutase, catalase and malondialdehyde, through activating the Akt/ERK1/2/Nrf2 signaling pathway (Yu et al., 2015). Tan ⅡA exerts robust antioxidant activity through the SIRT1 signaling pathway (Feng et al., 2016). It also reduces the accumulation of free radicals in radioactive brain injuries (Sun et al., 2017). Sal A is essential to protect cells from damage caused by toxic stimuli (Wang and Xu, 2005). Sal B protects against oxidative damage by upregulating the Nrf2 antioxidant signaling pathway, which may be regulated by activating the SIRT1 pathway (Zhang et al., 2018). Through angiotensin type I receptors, angiotensin II activates the reduced nicotinamide adenine dinucleotide phosphate, which results in the formation of ROS in the vasculature (Gonzaga et al., 2018). In this progress, Sal B also downregulates angiotensin type I receptors in the vessel wall to alleviate the deleterious effect of angiotensin II, an essential stimulant for the production of ROS in the vascular system (Ling et al., 2017).

### 4.4 Endothelial protective effect

The endothelium, formed by a single endothelial cell layer, lines all blood vessels, such as arterioles, venules and veins (Lee et al., 2022). The endothelium regulates blood homeostasis *via* controlling blood fluidity, continuity, and fibrinolysis (Triggle et al., 2012). Endothelial cells have proteins involved in the various functions of leukocytes (Panés and Granger, 1998). Dysfunction and altered structure of the endothelial layer during pregnancy are associated with PMPCs (Ghafourian et al., 2022). Danshensu protects endothelial cells *via* the CD40 pathway and inhibition of apoptosis by downregulating the proportion of cells in the G(0)/G(1) phase (Yang et al., 2009). In addition, it reduces the serum levels of homocysteine, a substance damaging endothelial cells (Yang et al., 2010). Tan ⅡA inhibits endothelial cell apoptosis by reducing the expression of related apoptotic proteins *via* the NF-κB signaling pathway, thereby exerting a protective effect on vascular endothelial cells (Liu et al., 2021). It also protects endothelial function through inhibiting endothelin-1 expression, decreasing endothelin type A receptors, increasing endothelin type B receptors, and upregulating eNOS (Chen et al., 2017). Cryptotanshinone’s endothelium protective action is mainly associated with the reduction of endothelial inflammation. In particular, cryptotanshinone blocks the scavenger receptor LOX1-mediated pro-inflammatory response in endothelial cells, preventing monocyte adhesion to endothelial cells (Li et al., 2018). Further researches are also necessary to determine the potential impact of cryptotanshinone on other crucial aspects of endothelium protection.

The above effects and mechanisms suggest that *S. miltiorrhiza* ameliorates adverse cardiovascular symptoms. Since PMPCs are characterized by insufficient blood perfusion, vascular endothelial dysfunction, and abnormal coagulation, *S. miltiorrhiza* and its active compounds can be applied in PMPCs (Bai et al., 2019). Thus, we profile the potential mechanisms of *S. miltiorrhiza* for PMPCs in Table 1.

## 5 Conclusion

In summary, PMPCs are a heterogeneous disease with similar mechanisms, including reduced trophoblast cell invasion and insufficient spiral artery remodeling, which results in placental hypoperfusion, endothelial dysfunction, and abnormal coagulation. *S. miltiorrhiza* effectively attenuates the symptoms of PMPCs through anticoagulation, vasodilation, inhibition of free radical formation, and protection of endothelial cells. Notebly, *S. miltiorrhiza* and its active compounds have been shown to treat PE, mitigate the severity of FGR, and improve the adverse symptoms of RSA. Hence, *S. miltiorrhiza* may be used to improve the pregnancy outcomes of pregnant women with PMPCs effectively. However, the specific effects of *S. miltiorrhiza* on PMPCs still need clinical verification, although animal models have provided much more valuable clues. *In vitro* and *in vivo* studies are required to clarify the related signaling pathways of. New techniques are needed to study human placental development and provide optimal therapy for patients with PMPCs.
